# Elucidating bacterial adhesion to mucosal surface by an original AFM approach

**DOI:** 10.1186/s12866-021-02303-1

**Published:** 2021-09-06

**Authors:** Karen Dunker, Sol Gomez de la Torre Canny, Catherine Taylor Nordgård, Etienne Dague, Cécile Formosa-Dague, Ingrid Bakke, Marit Sletmoen

**Affiliations:** 1 Department of Biotechnology, NTNU Norwegian University of Science and Technology, NO-7491 Trondheim, Norway; 2 LAAS-CNRS, Université de Toulouse, CNRS, 31400 Toulouse, France; 3 TBI, Université de Toulouse, CNRS, INRAE, INSA, 31400 Toulouse, France

**Keywords:** AFM, Mucin, Mucous, Glycobiology, Glycan, bacterial adhesion, salmon

## Abstract

**Background:**

Fish skin represents an ancient vertebrate mucosal surface, sharing characteristics with other mucosal surfaces including those of the intestine. The skin mucosa is continuously exposed to microbes in the surrounding water and is therefore important in the first line defense against environmental pathogens by preventing bacteria from accessing the underlying surfaces. Understanding the microbe-host interactions at the fish skin mucosa is highly relevant in order to understand and control infection, commensalism, colonization, persistence, infection, and disease. Here we investigate the interactions between the pathogenic bacteria *Aeromonas salmonicida (A. salmonicida)* and *Yersinia ruckeri (Y. ruckeri)*, respectively, and the skin mucosal surface of Atlantic salmon fry using AFM force spectroscopy.

**Results:**

The results obtained revealed that when retracting probes functionalized with bacteria from surfaces coated with immobilized mucins, isolated from salmon mucosal surfaces, rupture events reflecting the disruption of adhesive interactions were observed, with rupture strengths centered around 200 pN. However, when retracting probes functionalized with bacteria from the intact mucosal surface of salmon fish fry no adhesive interactions could be detected. Furthermore, rheological measurements revealed a near fluid-like behavior for the fish fry skin mucus. Taken together, the experimental data indicate that the adhesion between the mucin molecules within the mucous layer may be significantly weaker than the interaction between the bacteria and the mucin molecules. The bacteria, immobilized on the AFM probe, do bind to individual mucins in the mucosal layer, but are released from the near fluid mucus with little resistance upon retraction of the AFM probe, to which they are immobilized.

**Conclusion:**

The data provided in the current paper reveal that *A. salmonicida* and *Y. ruckeri* do bind to the immobilized mucins. However, when retracting the bacteria from intact mucosal surfaces, no adhesive interactions are detected. These observations suggest a mechanism underlying the protective function of the mucosal surface based on the clearing of potential threats by adhering them to loosely attached mucus that is subsequently released from the fish skin.

## Background

Mucus secretions are a central protective barrier covering the epithelium and found over the whole class of metazoans. Despite this protective function, the mucosa is the access point for the majority of human pathogens. Successful infection depends on the ability of the microbes to move through the mucus in order to attach to the underlying cells. Mammals have mucosal tissues in the gastrointestinal (GI), respiratory, reproductive, and urinary tracts as well as mucosa coating the eye. Fish have a skin mucosa in addition to the mucosa present in mammals; the skin mucosa works as the fish’s first line of defense against microbes [1]. The barrier function of mucus layers can be attributed to three distinct classes of underlying mechanisms: (i) mechanical barrier function: the mucin hydrogel prevents entry of particles exceeding a certain size, (ii) interactive barrier function: mucins mediate adhesion of microbes through their protein backbone and/or oligosaccharide side chains, retaining the microbes in the mucosal layer and (iii) dynamic barrier function; involving a continuous mucus secretion and internal mucin dynamics, resulting in efficient removal of microbes trapped in the mucus layer. The dynamic barrier properties are influenced mainly by mucin structure and overall mucus architecture [2, 3].

Mucins are the main protein component of mucus, and they are characterized by being heavily O-glycosylated. Mucins carry a variety of glycan structures and O-glycans can account for up to 50–80% of the total mass of the molecules [1]. The presence of negatively charged glycans, containing sialic acid units for example, give the molecules a net negative charge. The sialic acids can be utilized by pathogens as nutrients [4] or attachment sites for bacterial adhesion [5]. The study of sialic acid binding adhesins has revealed similar lysine-rich sialic acid binding motifs in lectins on *Escherichia coli* and *Helicobacter pylori* [6, 7]. A large diversity in mucin glycosylation is observed between species, and also between organs within the same animal [8]. This diversity is previously suggested to enable the host to facilitate adhesion of specific microbes as observed in the gut [9].

For most bacteria the molecular mechanisms underlying their adhesion to mucosal surfaces are complex and not fully elucidated. Multiple mechanisms may play a part, and the relative importance of each mechanism may change over time and between different adherent surfaces [10] making the identification of individual adhesion mechanisms challenging. Primary adhesion occurs when the bacteria are brought into proximity of the surface through active or passive movement. The adhesion strength is determined by the sum of repulsive and adhesive forces. Interactions that should be taken into consideration when aiming to understand bacterial adhesion include electrostatic and hydrophobic interactions, steric hindrance, van der Waals forces and hydrodynamic forces. Furthermore, the adhesion may be due to both non-specific interactions and specific ligand-receptor interactions. The description of the non-specific interactions is strongly inspired by physicochemical approaches with DLVO theory, named after the researchers Derjaguin, Landau, Verwey and Overbeek and explaining the forces acting between charged surfaces interacting through a liquid medium [11–14]. The specific interactions include but are not limited to glycan-lectin interactions. Interestingly, the body of evidence documenting that glycans engage in direct interactions with other glycans is increasing [15]. Glycans are an important component of both the glycocalyx of cells mucosal surfaces as well as the surface of most bacteria [16]. The existence of glycan-glycan interactions opens for new mechanisms of bacterial adhesion to biological surfaces including mucosal surfaces, as documented in recent publications [17, 18]. The role of glycan-glycan interactions in bacterial adhesion has also been reviewed [15, 19].

The ability to adhere to the surface of host cells is important for bacteria since this is an essential step in bacterial pathogenesis or infection, required for colonizing a host [20]. It is presumed that microbial symbionts receive host-derived nutrients or a competition-free environment with reduced predation [21]. Microbial associations are integral to all eukaryotes, and mutualism, the interaction of two species for the benefit of both, is an important aspect of microbial associations. Oligosaccharide expression can provide a mechanism for selecting which bacteria can adhere to the host [22]. In other mucosal surfaces, such as the respiratory mucus, the oligosaccharides enhance the adhesion of a predefined group of bacteria, and facilitate their removal by mucociliary transport [23]. The array of oligosaccharides expressed on the mucins of an individual may therefore play a key role in governing the susceptibility to infection [12]. Padra and colleagues found that after a two-hour incubation time, *Aeromonas salmonicida* was present in a higher density on surfaces coated with mucins from Atlantic salmon skin as compared to the gut. Removal of sialic acids decreased binding to gut mucins and completely removed binding to skin mucins [24]. Linden and colleagues have also previously characterized the glycosylation of salmon mucins and found that salmon O-glycans are heavily sialylated with a different sialylation found on skin and gut mucins [10].

In the quest for improved understanding of bacterial adhesion mechanisms, atomic force spectroscopy (AFM) has proven to be a powerful technique. The mode of action of an AFM instrument has been described previously [25]. In addition to its use as an imaging tool, AFM can also be used in a force-distance (F-D) based mode (force-spectroscopy) to measure the interactions between the AFM cantilever and a surface. Cells can be attached to the AFM cantilever and brought into contact with a functionalized surface before being retracted, enabling the quantification of adhesive forces between the attached cell and the surface with a precision down to a piconewton scale [26]. The major advantages of AFM over other imaging techniques is that it operates in liquid setup making it possible to study live samples. Moreover, it requires little sample preparation, and the samples can be kept at physiological pH and room temperature over the course of an experiment [27].

This study investigated the adhesion of two common salmonid fish pathogens, *Yersinia ruckeri* and *A. salmonicida* [28] to Atlantic salmon yolk sac fry skin mucosa and to mucins isolated from the skin and proximal intestine of adult Atlantic salmon using AFM. Additionally, rheological methods are utilized to better understand the physical properties of the skin mucus. Finally, the effect of sialic acids in the pathogen – mucin interactions were investigated by comparing the adhesion of the bacteria prior to and after the addition of neuraminidase to the mucins. Based on the totality of the results a model explaining the protective mechanism of the mucosal surface of the fish fry is proposed.

## Results

### Immobilization of bacteria onto AFM probes

**Fig. 1:**
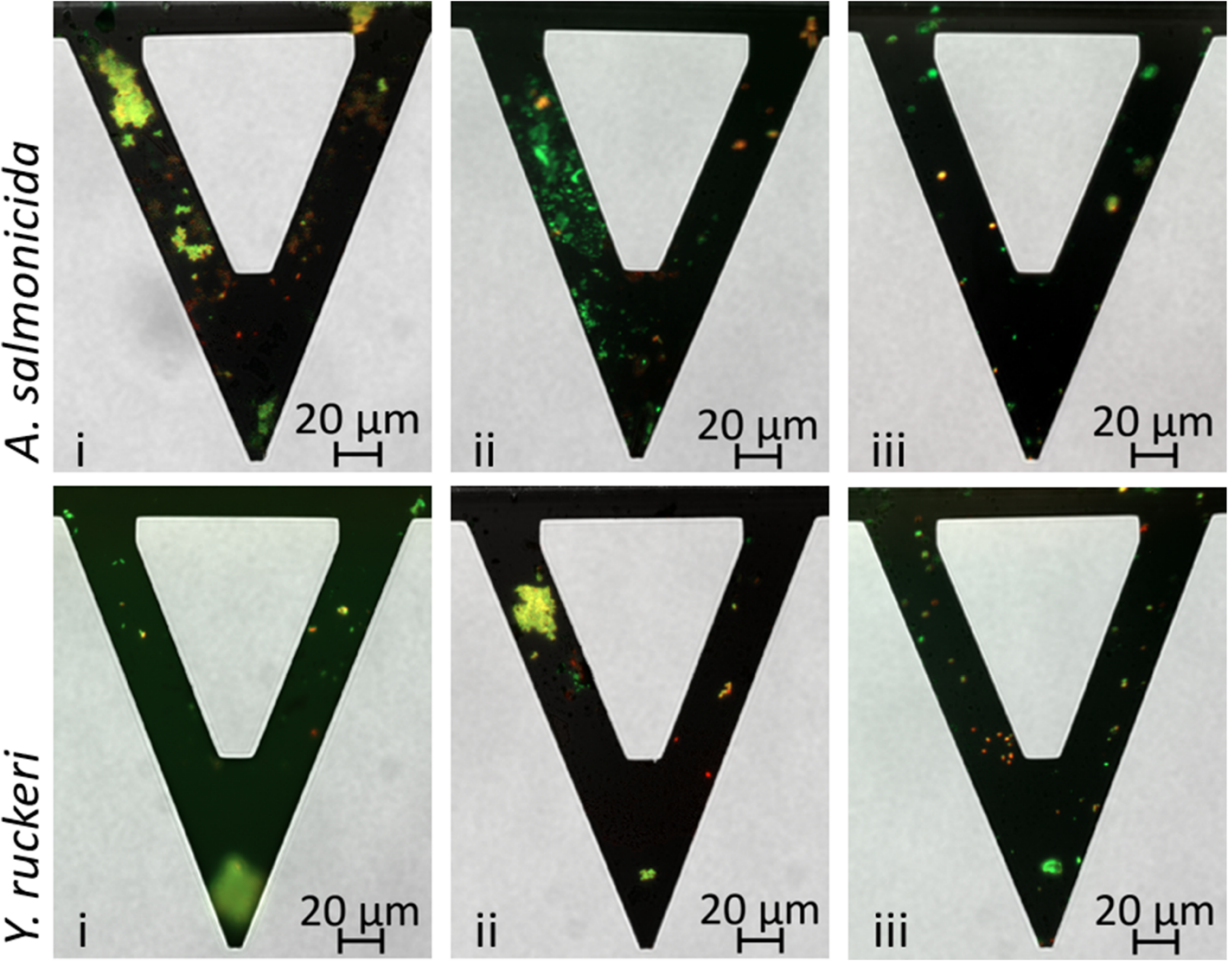
Representative microscopy images of PD-coated AFM cantilevers onto which bacterial cells had been immobilized. The probes shown were obtained by leaving the AFM probes in contact with the solution containing the bacteria for a duration of 12 h. Images were obtained on a Zeiss Axio Observer.Z1 with 20 x magnification

The bacteria were immobilized on PD-coated AFM probes. Inspection of the functionalized probes using light microscopy revealed that the density of immobilized bacteria increased with increasing contact time between the PD-coated probes and the suspension containing the bacteria (data not shown). A contact time equal to 12 h was used. For most of the probes this resulted in an acceptable density of immobilized bacteria (Fig. 1). Figure 1 shows AFM probes with immobilized bacteria after a 12 h contact time. A live/dead assay was used to illustrate the viability of the bacteria. Green fluorescence indicates live cells, while red fluorescence indicates dead cells.

The frequency of adhesive events between the AFM probe and the functionalized surface is influenced by the density and position of the immobilized bacteria on the probe. To further evaluate the probe preparation method, the frequency of adhesive events for the different probes was determined by counting the number of force-distance curves that displayed a force-jump (Table 1). The table shows lower frequency of adhesive events for the *A. salmonicida* probe iii compared to probe i and ii. Probe iii also has a lower surface coverage of bacteria on the cantilever tip compared to what is observed for probe i and ii (Fig. 1). For the rest of the cantilevers, a consistent high frequency of adhesive events was observed.

**Table 1:** Frequency of adhesive events between *A. salmonicida* and *Y. ruckeri* bacterial probes and mucin-coated surfaces. Parallels i-iii represents three separate bacterial probes used against three separate mucin-coated surfaces. MucS and MucI are mucins isolated from the skin and proximal intestine, respectively, of adult Atlantic salmon individuals

	Frequency (%)	
Parallel	MucS	MucI
***A. salmonicida***		
i	93.2	70.1
ii	86.0	90.4
iii	47.3	42.5
Mean ± std	75.5 ± 20.2	67.7 ± 19.6
***Y. ruckeri***		
i	99.9	99.2
ii	91.9	97.1
iii	87.7	95.9
Mean ± std	93.2 ± 5.1	97.4 ± 1.4

### Quantification of bacterial adhesion to the mucosal surface on fish fry

**Fig. 2:**
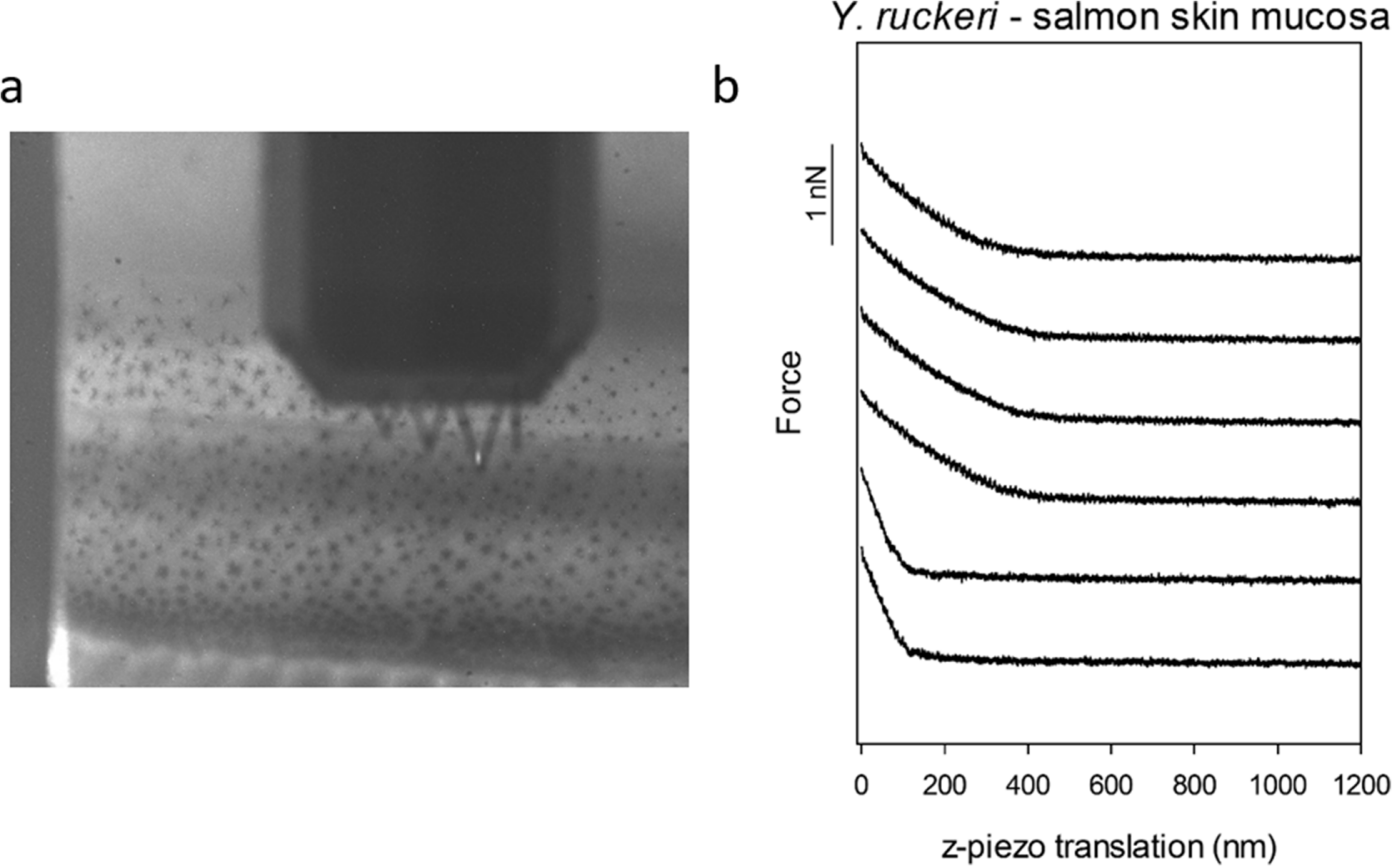
Bacterial adhesion to salmon skin mucsoa. **a**) Image showing a salmon sample under the AFM probe. The image was recorded using the built-in CCD camera on the ForceRobot 300. **b**) Representative force-distance curves showing the interaction between bacterial probes and the skin mucosal surface of salmon fry 8 weeks post hatching (WpH)

Adhesion between AFM probes functionalized with *A. salmonicida* and *Y. ruckeri*, respectively, and the skin mucosal surface of Atlantic salmon fry were investigated using AFM force spectroscopy. Salmon fry 8 weeks post hatching were immobilized and placed on the AFM to elucidate how bacteria interact with the intact biological surface (Fig. 2a).

As illustrated by the force-distance curves in Fig. 2b, no adhesive interactions were detected between the probe functionalized with bacteria and the skin mucosa. Furthermore, the steep rise in force at a short z-piezo translation distance revealed that contact with the surface was achieved, and the probes did indent a short distance into the mucosal surface, but no adhesive interactions are observed upon retraction. This behavior was consistent across the skin surface and between bacterial probes.

### Rheological measurements

**Fig. 3:**
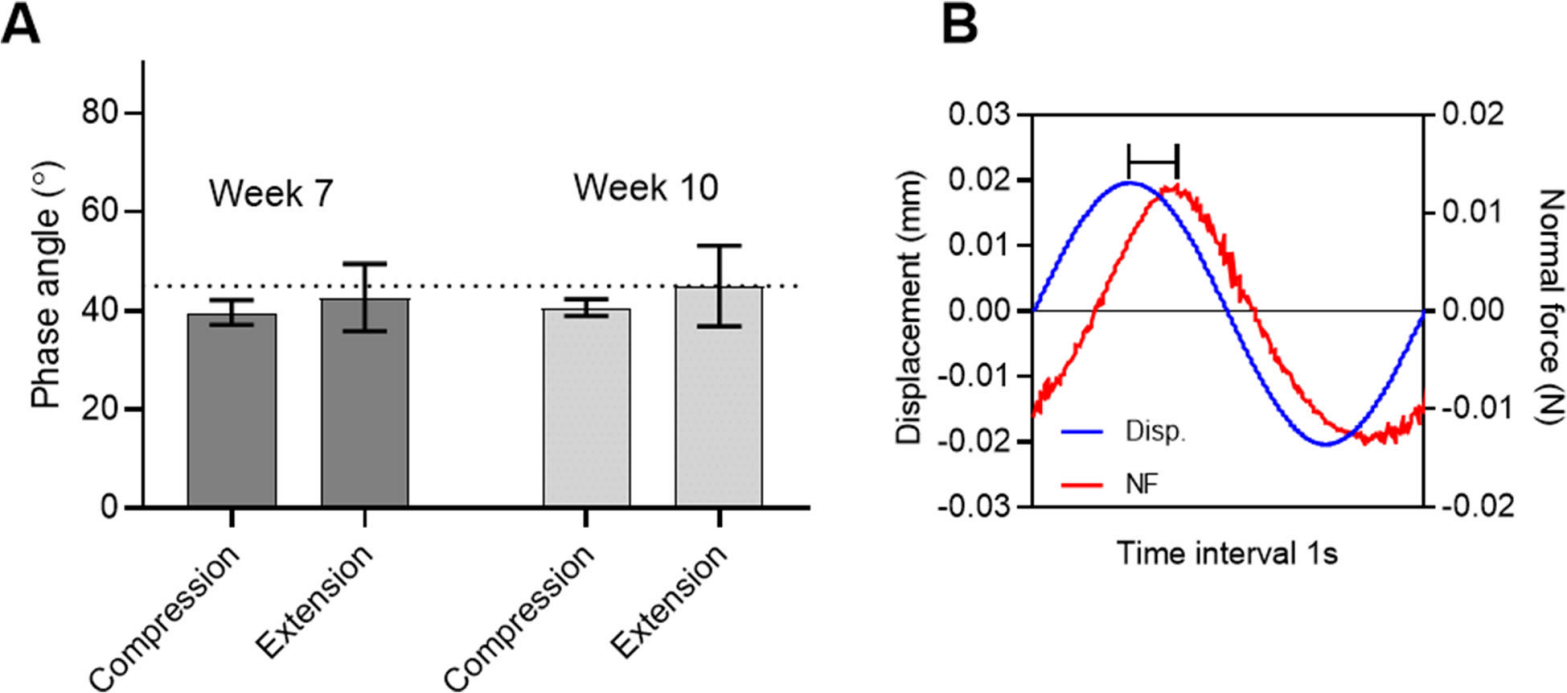
Rheological behavior of the outer layer of salmon fry mucosa. **A**) mean +/− S.D. of the phase angle for mucus from 3 individual fish between maximum displacement and force in compression and extension at week 7 and week 10 (the dashed line represents a phase angle of 45°, where elastic and viscous contributions to rheology are equal in magnitude) and **B**) an example of a single deformation cycle showing the applied displacement and the normal force response with the phase shift highlighted

The outer mucosal surface of the fish fry, the mucus layer, is composed of mucins, but these mucins are contained within a dynamic viscoelastic gel rather than being immobilized to a solid surface. As such, the lack of adhesive interactions between the bacteria and the mucosal surface of the fish fry could be due either to a lack of interactions coupling the different molecules in the outer layer of the mucus surface to the surface of the bacteria (scenario 1) or a lack of interactions between the different molecules within the outer layer of the mucus surface resulting in low mucus cohesivity and force-free removal of the molecules of the mucosal surface that have become attached to the bacteria (scenario 2). In order to identify which of these two scenarios that best describe the system, rheological experiments were performed on mucus removed from the skin by suction. The mucus showed rheology typical of a viscoelastic material. The mean phase angle indicated a material where the elastic behavior was only slightly dominant over the viscous behavior, with some individual measurements showing viscous dominant behavior (Fig. 3). A phase angle of 0° represents purely elastic behavior, whereas 90° represents purely viscous behavior and 45° equal contributions from elastic and viscous behavior. The compression and extension parts of the oscillatory cycle were not equivalent, and the phase angle in extension was slightly higher than in compression for both time points.

The phase angles measured here are indicative of a material which flows readily with relatively little cohesive elastic resistance, potentially making the measurement of attachment forces difficult. For comparison the phase angle of saliva has been reported to be around 20° (corresponding to an elastic response around 3 times larger than the viscous response) [29] and a similar phase angle for saliva was measured with the rheological set up used here (data not shown). These data support scenario 2 described above.

### Deadhesion work associated with detachment of bacteria from surfaces coated with mucins isolated from the skin and gut of salmon

The work required to rupture the adhesive interaction (i.e. the deadhesion work) formed between the two pathogens *A. salmonicida* and *Y. ruckeri* and mucins derived from Atlantic salmon were measured using AFM force spectroscopy. Mucins isolated from the skin (mucS) and proximal intestine (mucI) of adult Atlantic salmon individuals were immobilized onto separate mica slides and used in these experiments. In some experimental series, the immobilized mucins were treated with neuraminidase to remove sialic acid groups in order to identify their potential effect on bacterial adhesion. The glycan structures present on mucS and mucI have been characterized by Jin et al. They determined that mucS expresses shorter (2–6 residues, 33 different structures) and less diverse O-glycans than mucI (2–13 residues, 93 different structures). MucS has the three sialic acids Neu5Ac, Neu5Gc, and Kdn whereas mucI only has the sialic acid Neu5Ac [30].

The analysis presented in the following is based on three experimental parallels for each mucin-pathogen combination. A new probe was used for each parallel and a total of 900 force-distance curves was recorded on each mucin-coated surface.

**Fig. 4:**
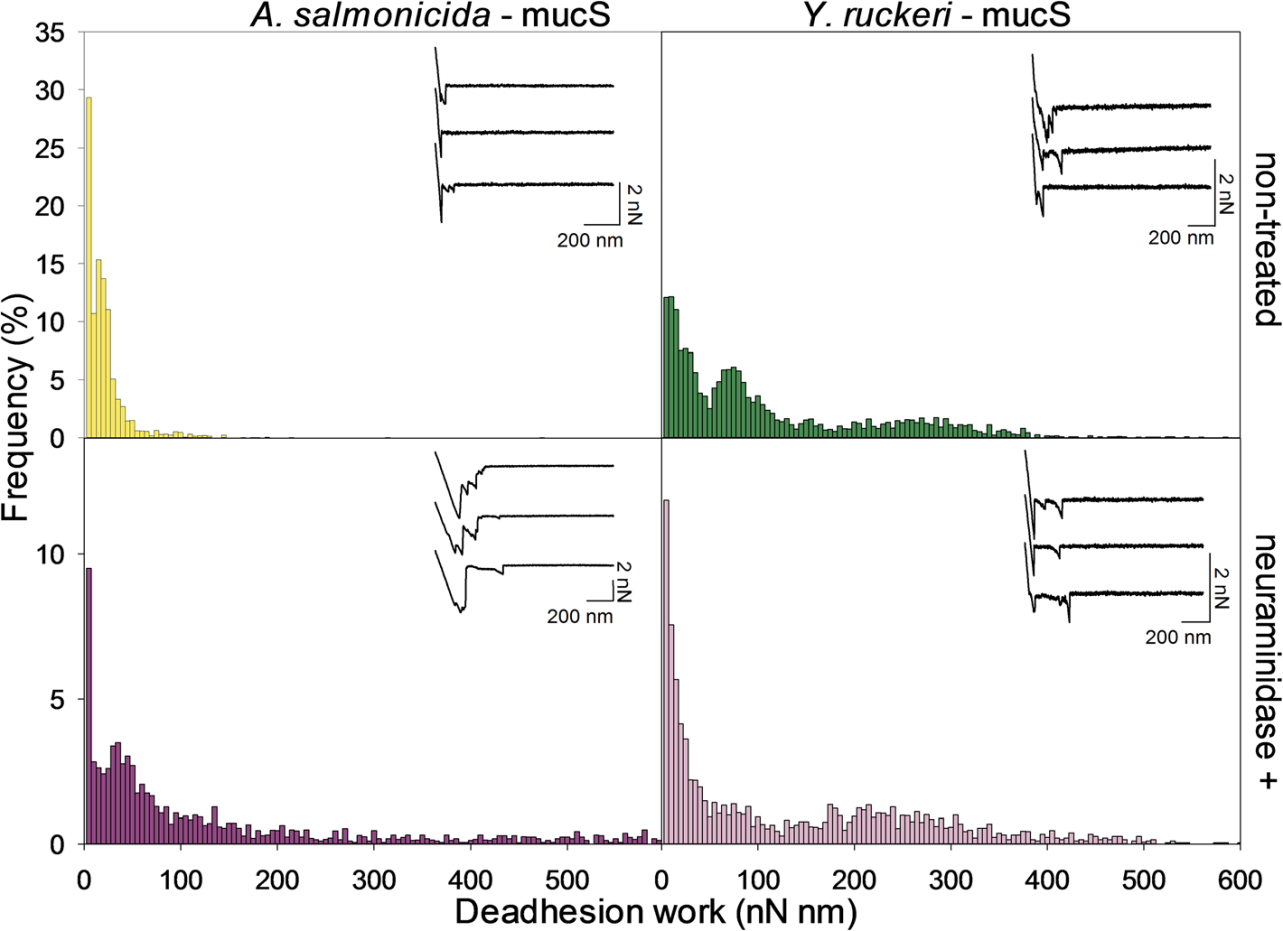
Deadhesion work for interactions between *A. Salmonicida* and *Y. ruckeri* and skin mucins of Atlantic salmon before and after neuraminidase-treatment of mucins. Three experimental parallels were performed with both pathogens, using three different mucin-coated surfaces and three bacterial probes. The analysis is based on 900 force-distance curves recorded on each mucin-coated surface. The histograms display the frequency (%) of magnitudes of deadhesion (nN mm) for the interactions measured with AFM force spectroscopy. Inset: representative force-distance curves obtained when quantifying the interactions between the bacterial probes and the skin mucin samples

**Fig. 5:**
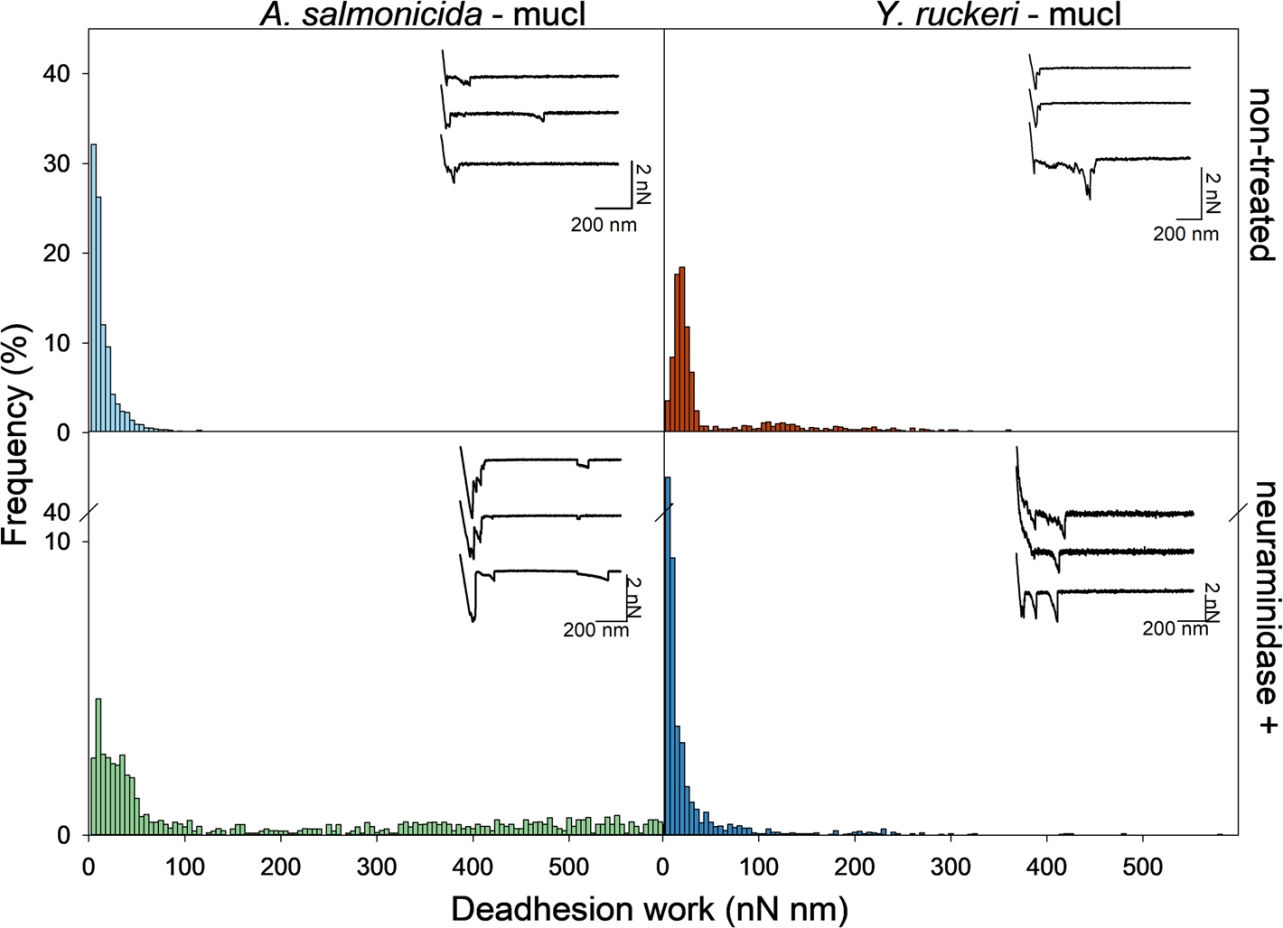
Deadhesion work for interactions between *A. salmonicida* and *Y. ruckeri* and proximal intestinal mucins of Atlantic salmon before and after neuraminidase-treatment of mucins. Three experimental parallels were performed with both pathogens, using three different mucin-coated surfaces and three bacterial probes. The analysis is based on 900 force-distance curves recorded on each mucin-coated surface. The histograms display the frequency (%) of magnitudes of deadhesion work (nN nm) for the interactions measured with AFM force spectroscopy. Inset; representative force-distance curves obtained when quantifying the interactions between the bacterial probes and the proximal intestinal mucin samples

The analysis of the deadhesion work, as revealed by the force-distance curves, revealed that both *Y. ruckeri* and *A. salmonicida* showed stronger adhesion to skin mucin than intestinal mucins (Figs. 4 and 5). Also, in the case of skin mucins, the neuraminidase treatment led to increased deadhesion work (Fig. 4). A similar increase is not observed in the case of *Y. ruckeri* and intestinal mucins, where the deadhesion work remains low after neuraminidase treatment. However, *A. salmonicida* showed a stronger adhesion to mucI after neuraminidase treatment (Figs. 4 and 5). Removal of sialic acid units also led to an increased probability for multiple interactions between *A. salmonicida or Y. ruckeri* and mucS, as illustrated by the multiple rupture events observed in some of the force curves (Fig. 4). Our observations indicate that *A. salmonicida* and *Y. ruckeri* show a higher tendency to bind to mucins originating from skin, as compared to intestines. Furthermore, the sialic acid does not seem to be essential for the initial interaction with the skin mucins, on the contrary the probability for interaction increases after removing the sialic acid unit. Since both *A. salmonicida* and *Y. ruckeri* are pathogenic bacteria, the data indicate that the sialic acid found on skin mucins contribute to reducing the initial adhesion of these pathogenic bacteria and by doing so it protects the fish against disease.

### Quantification of the strength of single adhesive interactions between mucins and their adhesin on the surface of bacteria

The force – z-piezo retraction curves obtained when retracting the bacteria from mucin-coated surfaces contained signatures of both stretching of mucin polymers and forced rupture between mucins and their binding partner(s) on the surface of the bacterium. The rupture events occurred at tip-surface separations in the range of 200–500 nm, which is compatible with the length of the mucin polymers. Many of the force – z-piezo retraction curves revealed several rupture events whereas others contained only one such event. Multiple rupture events may occur either because a single mucin chain is attached to several separate binding partners or because several mucin molecules interact with different binding partners. In the analysis of the rupture strengths, only well separated force jumps, where the spring returned to the base line after bond rupture, were included.

**Fig. 6:**
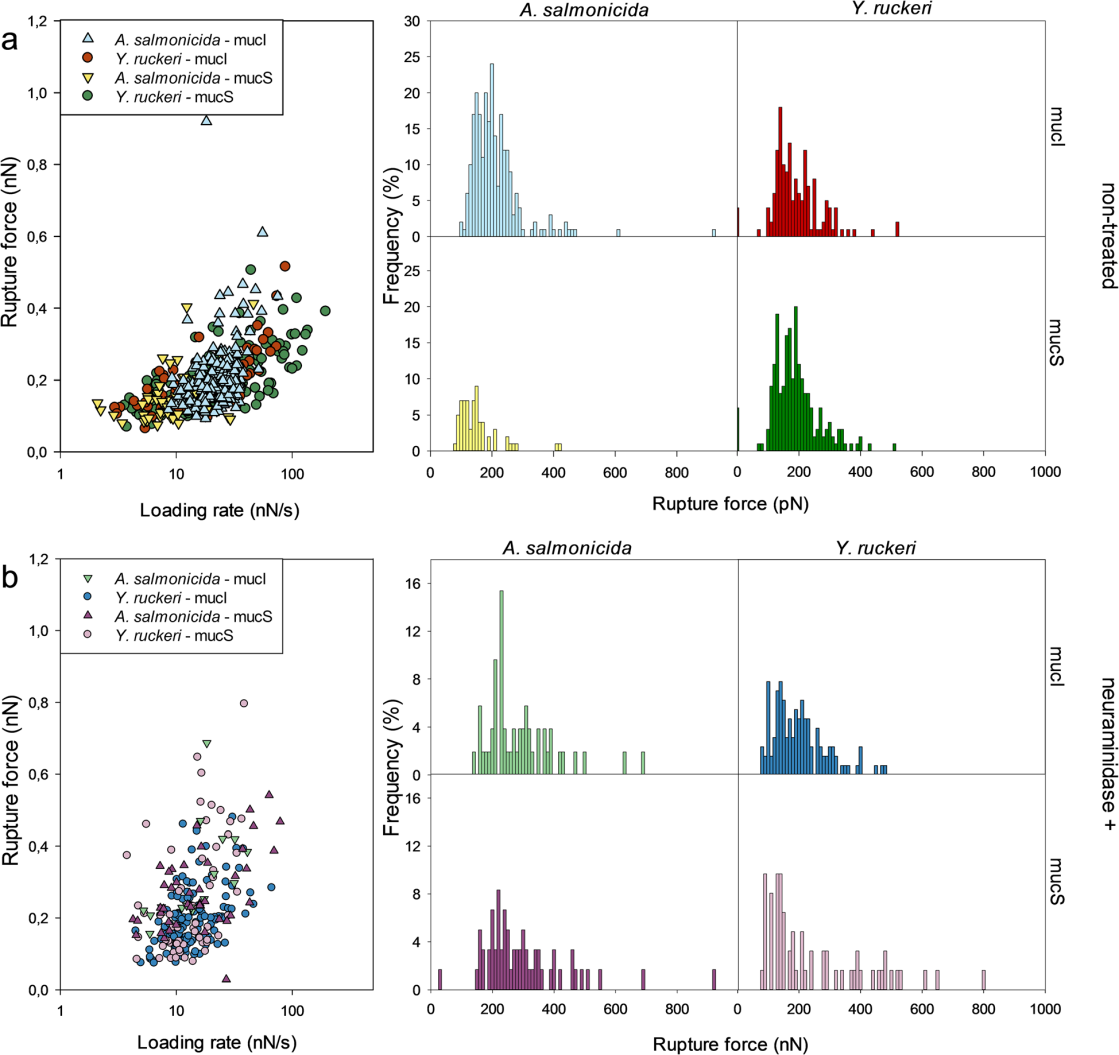
Rupture force for interactions between *A. Salmonicida* and *Y. ruckeri* and proximal intestinal mucins (mucI) and skin mucins (mucS) of Atlantic salmon before (**a**) and after (**b**) neuraminidase-treatment of mucins. The scatter plots show the rupture force against loading rate. The histograms display the frequency (%) of magnitudes of rupture force (nN) for the interactions measured with AFM force spectroscopy

The main portion of the rupture events give rupture forces in the interval 50 piconewton (pN) - 300 pN. No clear difference in rupture force was observed between *A. Salmonicida* and *Y. Ruckeri* when interacting with either mucI or mucS. However, for *A. Salmonicida* bacteria the distribution of rupture forces is shifted towards weaker forces when interacting with the skin mucins compared to the proximal intestinal mucins. The same trend is also observed for *Y. Ruckeri*, even though the shift in rupture forces for this bacterium is less pronounced (Fig. 6a). This can either be explained by a higher contribution from multiple interactions in the dataset obtained for MucI, or the interactions formed between the bacteria and their binding motive on MucI are stronger.

Figure 6b presents data obtained after removing sialic acid units by neuraminidase treatment of the mucins. When allowing the bacteria to interact with MucS, the distribution of rupture forces is, after neuraminidase treatment, shifted towards higher values. This is in accordance with the increased fraction of multiple interactions observed in the force curves (Figs. 4 and 5). This tendency is not observed when looking at the data obtained for MucI, in the sense that no change in rupture forces are observed after the neuaminidase treatment. These observations are in accordance with the data obtained for the deadhesion work (Figs. 4 and 5), in the sense that the neuraminidase treatment also did not influence on the deadhesion work.

## Discussion

Several studies published over the last decades document that AFM can reliably be used to study how bacteria interact with mucosal surfaces [10, 31–33]. In the present study we demonstrate that AFM can also be used to investigate the interactive surface properties of an intact animal [34], as we demonstrate the ability to place an intact salmon fry in the AFM. Mucosal surfaces are known to have a complex molecular organization that underlies many of their properties, including a layered structure where consecutive layers differ in molecular composition and functional properties [5]. The ability to investigate mucosal surfaces with the high resolution offered by the AFM is therefore important since this technique does not require the removal of the mucosal surface from the animal, minimizing the risk of introducing changes in molecular organization and functional properties during sample preparation.

The results obtained in the current study revealed that no adhesive interactions could be detected when retracting bacteria from the intact mucosal surface of salmon fry. Both *A. salmonicida* and *Y. ruckeri* probes were tested against the skin mucosa of conventionally reared salmon fry (CVR). Little to no adhesion was observed for the salmon samples. An important function of the mucosal surfaces is to form a barrier to pathogens present in the external environment of the fish, preventing these bacteria from accessing the underlying surfaces [33, 35]. A lack of adhesion between the bacterium and the mucosal surface was detected.

However, if immobilizing the mucins contained in mucosal surfaces on fish skin and fish gut to mica, rupture events reflecting the rupture of adhesive interactions were observed, with rupture strengths centered around 200 pN. This strength is in the same range as previously observed in AFM studies of the adhesion of *Lactococcus lactis* interacting with PGM, where the rupture strength was determined to be 180 pN [10]. Adhesion strengths in this range typically describes single molecular pair interactions [15]. This indicates that the bacteria studied do bind to mucins extracted from salmon skin and intestines through interactions between adhesins on the surface of the bacteria and structures on the mucin molecules. The precise identity and specificity of these adhesins is still unknown. As mentioned in the introduction, the number of examples of bacteria that adhere through interactions between two glycan structures is increasing. This mechanism of adhesion should therefore be considered as a likely, yet underexplored, alternative to glycan – protein, protein – protein or other alternative mechanisms of specific adhesion.

Phase angle measurements revealed a near fluid-like behavior for the mucus in the developmental stage investigated in the current study. This information in combination with information published in previous studies of bacterial adhesion to mucosal surfaces helps to explain why so little adhesion was detected between the bacteria and the intact mucosal surface. The observed lack of rupture events between bacterial probes and the mucosal surfaces of the fish fry can be explained by one of the two following scenarios. First, the bacteria could be unable to bind to the mucin structures at all, enabling retraction of the probe with no resistance. However, our observed interaction between the bacteria and the immobilized mucins is not in accordance with this hypothesis. Second, the adhesion between the different mucin molecules within the gel can be significantly weaker than the interaction between the bacterium and the mucin molecule, meaning that bacteria bind to individual mucins that will then be released from the fluid mucus with little resistance. This latter scenario resembles the situation previously described in a study on *H. pylori* in mice by McGuckin and colleagues. They demonstrated that muc1 binds to *H. pylori* and functions as a decoy, preventing the bacterium from sticking to the underlying surface. The animals with high muc1 expression bound more *H. pylori* to their mucins but had lower rates of infection [36]. The adhesive bond formed between the bacterium and the loosely bound mucin results in a scenario where the mucin is released and takes the bacterium with it, effectively clearing it from the system [37]. Skin mucus in fish has a high turnover rate [38], making this potential mechanism of bacterial clearance efficient.

The results obtained in the phase angle measurements performed as part of the present study support the low internal adhesion between mucins in the mucosal structure, and these data therefore further strengthen the hypothesis presented above. The recorded phase angle values are consistent with the surface mucus layer showing behavior that is only slightly elastic dominant at this developmental stage for the salmon fry. The mucin molecules in the mucosa appear to have only weak adhesion to each other. As a result, mucins may bind to the bacteria, but they will be easily released from the mucus layer during retraction of the probe onto which the bacterium is bound. In nature, bacteria adhering to the mucins will be prevented from forming attachments to the underlying surface and this hampers infection.

Salmon fry are in direct contact with the environment after hatching and are amenable to be colonized by the aquatic microbial populations. They are surrounded by water containing bacteria that can cause disease if able to enter their interior and are required to have an efficient protective barrier in order to survive. From this perspective, the low adhesion we observed is consistent with a functional mucosa that clears potential threats from the fish skin. However, previous studies have confirmed that fish skin mucosa has a commensal skin microbiota with a different composition than the microbiota of the surrounding water [39, 40]. This indicates that some bacteria behave differently from the pathogenic bacteria studied in the present study in the sense that they are capable of adhering to the mucus and remaining there. The host has the capacity to change its glycosylation pattern in response to an increased bacterial load and commensal bacteria have also been shown to modulate glycosylation [41]. As the glycans are crucial in forming adhesion to the mucins, this adaptation can provide a mechanism for the host to select for certain members of the bacterial community [9].

There are several potential explanations for the low bacterial adhesion to the skin mucus observed in the present study. The fish fry studied here were in an early developmental stage. The fry could have a less complex glycosylation or a mucosal structure unique to this life stage. Yolk sac fry have a microbiota that deviate from this of older fish [42]. Salmon are potentially more vulnerable to infection when they are younger due to their thinner skin, and it could therefore be even more crucial to keep bacteria away from the skin at the early life stages compared to later stages. Alternatively, the fish select for beneficial bacteria over pathogenic ones by modulation of the mucus layer, as described in a review by Benhamed et al. [43]. Furthermore, previous studies with *A. salmonicida* performed on adult salmon revealed that fish with a thinner skin mucus were more prone to infection, but that wounds in the skin were required for the pathogen to gain access [44]. However, this should not strongly influence the initial adhesive events that are the focus of our study.

The AFM based investigation of mica surfaces coated with immobilized mucins isolated from salmon, as described in the present study, provided new insight into bacterial adhesion to the mucosal surfaces on salmon fry. Both pathogens included in this study, adhered more strongly to the mucin-coated surfaces than to the intact salmon skin mucosa. Furthermore, they adhered more strongly to the gut mucins as compared to skin mucins, a difference that might be explained by the different glycosylation that has been determined for these mucins [40]. However, the amount of adhesion observed was found to also depend on the density and location of the immobilized bacteria on the AFM probe. (Fig. 2, Table 1). This observation highlights the importance of both the AFM probe preparation method and the imaging of the bacterial probes prior to use in order to aid the interpretation of the adhesion data recorded. However, applying the exact same probe in investigations of different surfaces, as done in the present study, reduces the variability introduced by differences between probes. In studies looking at single-cell adhesion, these challenges related to bacterial density and position are circumvented [26].

Padra and colleagues demonstrated that N-Acetylneuraminic Acid (NAA) is important for the adhesion of *A. salmonicida* to salmon skin and gut mucins and the removal of NAA from the skin mucins resulted in total loss of adhesion [24]. These prior findings inspired us to investigate the effect of sialic acid removal on the adhesion of *A. salmonicida* and *Y. ruckeri* to immobilized salmon mucins. The results obtained in the present study are not consistent with the effect observed by Padra’s group, as the removal of sialic acids caused a slight increase in the adhesion of *A. salmonicida* and had no significant effect on *Y. ruckeri*. A potential explanation for this apparent discrepancy is that the current study focuses on the initial adhesion only, whereas Padra’s study looked at bacterial adhesion after a longer contact period where additional mechanisms may play a part. The negatively charged sialic acid units will create an electrostatic repulsion to the negatively charged surface of Gram-negative bacteria such as *A. salmonicida* and *Y. ruckeri.* Removing the sialic acid units leads to decreased negative surface charge. This might influence the probability that the bacterium will come in sufficiently close contact with the mucins for adhesive interactions to form. Furthermore, the lack of correlation between neuraminidase treatment and bacterial adhesion observed in the current study indicate that the sialic acid unit is not essential for the initial steps in the bacterial adhesion. However, the precise molecular binding partners involved in this bacterial adhesion are still not identified, and this lack of knowledge complicates the interpretation of the data. Our results demonstrating weak adhesion between *A. salmonicida* and *Y. ruckeri*, respectively, and mucins, as well as low internal adhesion of mucins in the mucosal layer are consistent with pre-existing knowledge about both pathogens. Experiences with pathogenic challenge has shown that it is difficult to induce infection in fish if the skin barrier is intact, and wounds are often a pre-requisite for the entrance of bacteria in the body [44, 45].

## Conclusions

The close contact between fish and microbes present in the surrounding water makes cutaneous diseases relatively common, and the skin mucosa is highly important in the first line of defense against environmental pathogens. For higher organisms, mucosal surfaces are important for protection of tissues and homeostasis but are often targeted by disease-causing bacteria. It is therefore important to increase the understanding of mechanisms underlying the efficient protection provided by mucosal surfaces. Still, these mechanisms are poorly understood and largely unexplored. In this study the interaction mechanism between the pathogenic bacteria *A. salmonicida* and *Y. ruckeri* and the mucosal surfaces of Salmon fry are explored. Our findings are consistent with a protective mechanism where the bacteria form adhesive interactions with the mucin molecules found in the mucous. However, rheological data obtained reveal that the surface mucus has a near fluid-like behavior. The bacteria are therefore likely to be shed from the mucosal surface together with the mucin molecules to which they are attached before they give rise to harmful effects. Furthermore, the results reveal that removal of sialic acid units leads to stronger interaction between *A. salmonicida* and both skin and proximal intestinal mucins, whereas the adhesion of *Y. ruckeri* is not significantly impacted. The lack of correlation between neuraminidase treatment and bacterial adhesion observed in the current study indicate that the sialic acid unit is not essential for the initial steps in the bacterial adhesion.

## Materials and methods

### Immobilization of bacteria on AFM probes

#### Cultivation of bacteria

*A. salmonicida* (strain V-88/09/03175 from the culture collection of the Central veterinary laboratory, Oslo, Norway) and *Yersinia ruckeri* (strain NVI – 11,025, isolated from diseased salmon, provided by the Veterinary Institute) were cultivated for immobilization onto AFM probes. The bacterial isolates were cultivated on tryptic soy agar based on TSB (Sigma Aldrich) with 15 g/L agar (VWR). Liquid cultures were grown in TSB at 21 °C for 24 (*Y. ruckeri*) or 48 (*A. salmonicida*) hours.

#### Functionalization of AFM probes

Dopamine hydrochloride (Sigma Aldrich) was dissolved in TRIS buffer (pH = 8.5 at 21 °C, Sigma Aldrich) to a concentration equal to 4 mg/ml, and allowed to polymerize on PNP-TR-TL tipless cantilevers (NanoAndMore GmbH) for 45 min. Probes were subsequently cleaned with MilliQ-water and carefully dried with filter paper. A volume equal to 50 μl of bacterial suspension was placed on top of the probe. *A. salmonicida,* and *Y. ruckeri* were immobilized on the probe after 12 h of incubation at 21 °C. After immobilization, the probes were rinsed with sterile salmon gnotobiotic medium (SGM) to remove any unattached bacteria.

#### Live/dead assays

The presence of immobilized bacteria on a probe was verified using live/dead staining and fluorescence microscopy. The L7012 LIVE/DEAD® Bac-Light Bacterial Viability Kit (Invitrogen) was prepared according to manufacturer instructions and the probes were imaged on a Zeiss Axio Observer.Z1 (excitation: 483 nm/305 nm). Probes were also imaged after completing the AFM measurements to verify that the bacteria stayed attached to the probe and remained alive.

### Preparation of mucin coated glass surfaces

Mucins from adult *Salmo salar* skin and proximal intestine were kindly provided by Sara Lindén (GU), and were isolated as described by Padra and coworkers [24]. The mucins were transferred from GuHCl to PBS (pH = 7.4 at 21 °C) through dialysis. The dialysis tubes were sterilized by dialysis against GuHCl for 12 h. The mucins were then transferred to the buffer through dialysis for 2 × 12 h against 1 M NaCl, 2 × 12 h against 0.5 M NaCl and 2 × 4 h against PBS. All dialysis was performed at 4 °*C. Mica* sheets were cleaved and functionalized with 3% N-[3-Trimethoxysilyl)propyl]ethylenediamine triacetic acid (abcr GmbH) in acetic acid (10 mM) for 20 min and rinsed with MilliQ-water. Salmon skin (mucS) and proximal intestinal mucins (mucI) were dissolved in boric acid (50 mM, pH = 5.5 at 21 °C) to a final concentration of 0.25 mg/mL with 0.5 mg/mL of EDC added as a coupling agent and applied to the mica slides. The reaction was allowed to continue for 1.5 h before removing the excess mucin solution and rinsing the mica sheets with MilliQ-water. The immobilized mucins were covered in salmon gnotobiotic medium (SGM) for the AFM measurements.

### Neuraminidase-treatment of mucins

Neuraminidase (from *A. ureafaciens*) was prepared according to manufacturer’s instructions (Sigma-Aldrich) and 4 units were added to each sample of the immobilized mucins. The mucins were incubated at 37 °C for 1 h before rinsing thoroughly with SGM.

### Atlantic salmon rearing

Fertilized eggs from Atlantic salmon (*Salmo salar L,* strain Aquagen) were provided by Aquagen AS (Trondheim, Norway) and hatched and raised in the dark at fish room (FRT, 6.5 °C). Whereas initially colonized with microbial communities from the hatchery and transport, yolk sac fry were maintained in sterile (autoclaved) salmon gnotobiotic media (SGM), a synthetic moderately hard freshwater (96 mg/l NaHCO_3_, 60 mg/l CaSO_4_•2H_2_O, 60 mg/l MgSO^4^•7H_2_O, 4 mg/l KCl), until sampling. Fish were maintained at a density of 15–18 fry in cell culture flasks (VWR) filled with 100 ml of sterile SGM. To maintain adequate water quality parameters for the optimal development of the animals, 60% of the SGM volume was exchanged every other day, and three times per week. Hatchability was used as the inclusion criterion for fish samples and death was used as the exclusion criterion. As all salmon fry included in this experiment were from the same treatment condition, randomization and blinding were not required.

### Preparation of fish samples

Yolk sac fry were euthanized with 0.52% (w/v) of MS-222 (tricaine) in SGM buffered with 1 M of Tris pH 9 (2.73:100 solution, final pH of 7.5) 8 weeks post hatching. Death was confirmed by the cessation of heartbeat under a stereomicroscope. Samples were rinsed in sterile SGM before immobilization. The salmon fry were then coated in 1% low-melt agarose gel (Sigma Aldrich), leaving the skin surface of the fish exposed. The gel was solidified by keeping the sample at 6 °C for 5 min. The samples were covered in SGM during AFM sampling. Fry were analyzed immediately after preparation.

### Quantification of bacterial adhesion to mucin coated glass surfaces or the surface of salmon fry using AFM

**Fig. 7:**
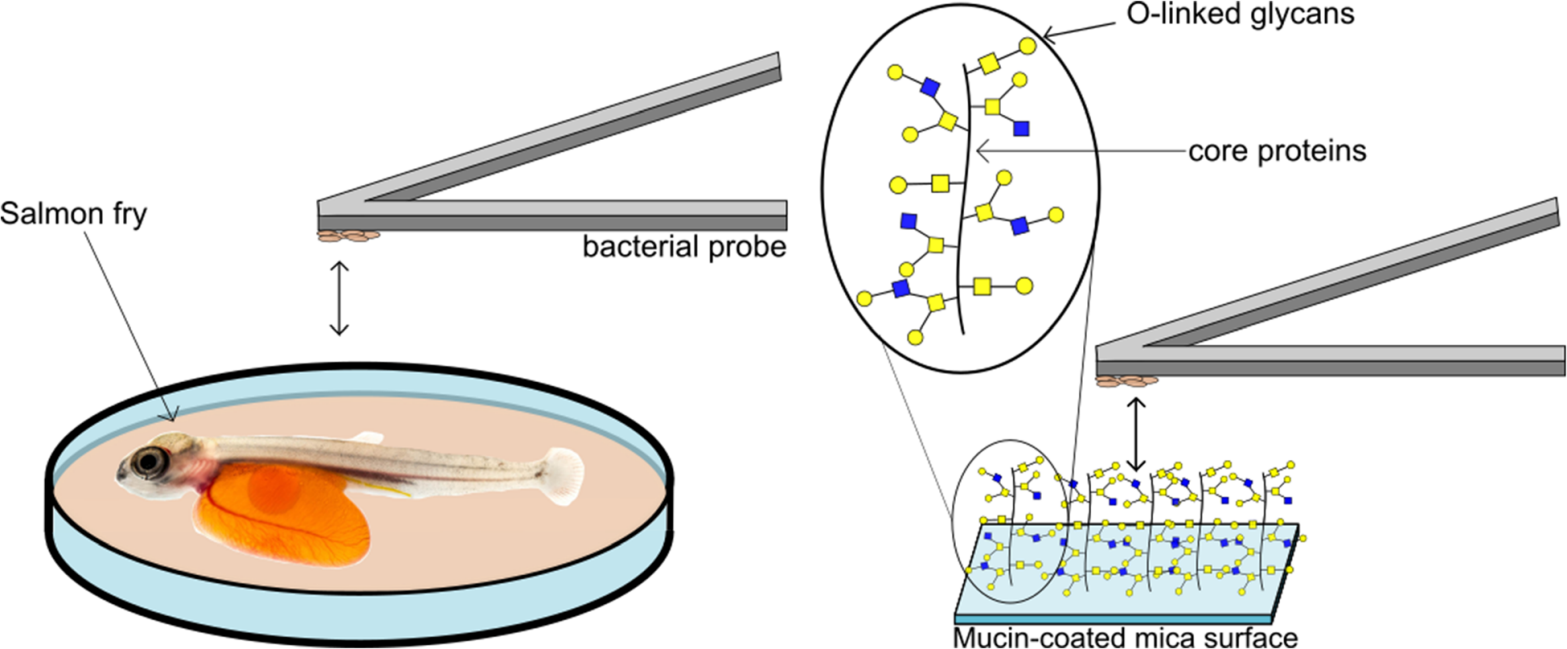
Schematic illustration of the experimental setup. a) AFM based investigations of bacterial adhesion to the skin mucosal surface on Atlantic salmon fry. The salmon fry were immobilized in an agarose gel. b) Experimental approach used when quantifying the adhesion strength between bacteria immobilized onto AFM probes and mucin proteins immobilized onto mica surfaces. All experiments were performed at room temperature (21 °C) in salmon gnotobiotic media (SGM)

AFM measurements were performed at room temperature (21 °C) in sterile SGM using a ForceRobot 300 spectroscope (Bruker, Berlin, Germany). The experimental setup is illustrated in Fig. 7.

Tipless cantilevers were calibrated using the thermal tune method in the ForceRobot software, giving spring constants equal to 0.048 ± 0.013 N/m for the *Y. ruckeri*-probes and to 0.047 ± 0.010 N/m for the *A. salmonicida*-probes. Force-curves were recorded across three separate areas of the mucin-coated mica surfaces with a z-length of 3–6 μm, a z-scan rate of 0.5 Hz and a maximum force of 1.14 nN. Force-curves were recorded across two different areas on the fish skin surface with a z-length of 15 μm, a z-scan rate of 0.5 Hz and a maximum force of 1.14 nN. Three fish were used with each probe. For each combination of probe and sample, at least 400 curves were recorded.

### Analysis of AFM data

The deadhesion work between the AFM probe and the mucosal surfaces were determined using the data processing software JPKSPM Data Processing (version spm-5.0.133). For the curves containing one or several force jumps, the area under the retract curve was determined and used to calculate the deadhesion work. The percentage of the curves containing a signature of interaction was also calculated (Table 1).

The AFM force curves containing one or several well-defined and separated rupture events were used as a basis to calculate the rupture force of single adhesive interactions between the bacterium and the mucosal surface. The rupture force was obtained based on the detected amount of deflection of the AFM cantilever prior to bond rupture, as revealed in the force curve, multiplied by the spring constant of the cantilever.

In the present study, force jumps observed in the force−distance curves were interpreted as evidence of the rupture of non-covalent interactions formed between mucin molecules immobilized to the sample surface or present on the surface of the fish, and adhesins present on the surface of the bacterium immobilized on the AFM probe. Since the mucin segments connect the interacting segments to the respective surfaces, the force loading rate does not only depend on the tip retraction velocity, but also on the length and properties of the polymer segments linking the two surfaces. The loading rate acting on an intermolecular bond was therefore determined for each force jump from the slope Δf/Δt prior to each observed dissociation event. The following rules were applied for the selection of force jumps: accepted jumps should only contain one peak and prior to deflection the cantilever should be in its resting position (i.e., not deflected). For each mucin sample investigated, the data obtained were displayed in dynamic force spectrum.

### Rheological studies

Mucus rheology was investigated as describedby Nordgard et al. [46]*.* A micropipette was used to gently remove skin mucus (70 μl per fish) from the body of individual freshly euthanized yolk sac salmon fry for rheological studies. A Malvern Kinexus Ultra+ rheometer fitted with 20 mm diameter parallel plates was operated in axial oscillation mode at 8 °C and a starting gap of 0.2 mm. A sequence was designed to sequentially run scripts to sinusoidally oscillate the normal force motor of the upper plate at a maximum strain amplitude of 10% (maximum displacement 0.02 mm) and a frequency of 1 Hz under constant temperature control while collecting raw data (gap and normal force) at a data rate of 200 points per second. From the gap and normal force data the phase shift between the applied deformation maxima and the measured force maxima were determined for both the compression and extension parts of the cycle and plotted as phase angle (degrees). Mucus from 3 fish at each time point was studied and mean data is presented alongside a typical raw data curve.

## Data Availability

The datasets used and/or analyzed during the current study are available from the corresponding author on reasonable request.
